# Preparation and application of fluorescent monoclonal antibodies recognizing goat CD4^+^CD25^+^ regulatory T cells

**DOI:** 10.1007/s00253-024-13115-4

**Published:** 2024-05-08

**Authors:** Yunpeng Wang, Haoyue Yang, Jiajin Hu, Yuecai Jiang, Wentao Ma, Shikong Gao, Dekun Chen

**Affiliations:** 1 College of Veterinary Medicine, Northwest A&F University, Yangling, Shaanxi China; 2 Shenmu Animal Husbandry Development Center, Shenmu, 719300 Shaanxi China

**Keywords:** Treg, CD4 mAb, CD25 mAb, *Orf*, Immunofluorescence staining, Flow cytometry

## Abstract

**Abstract:**

Regulatory T cells (Tregs) are a subset of T cells participating in a variety of diseases including mycoplasmal pneumonia, contagious ecthyma, and so on. The role of Tregs in goat contagious ecthyma is not completely understood due to the lack of species-specific antibodies. Here, we developed a combination of CD4 and CD25 fluorescence monoclonal antibodies (mAb) to recognize goat Tregs and assessed its utility in flow cytometry, immunofluorescence staining. Using immunofluorescence staining, we found that the frequency of Treg cells was positively correlated with the viral load during *orf* virus infection. These antibodies could serve as important tools to monitor Tregs during *orf* virus infection in goats.

**Key points:**

*• A combination of fluorescent mAbs (C11 and D12) was prepared for the detection of goat Tregs.*

*• C11 and D12 are effective in flow cytometry, immunofluorescence staining, and C11 has excellent species specificity.*

*• The frequency of Treg cells was positively correlated with the viral load during orf virus infection.*

**Supplementary information:**

The online version contains supplementary material available at 10.1007/s00253-024-13115-4.

## Introduction

CD4^+^CD25^+^ Tregs have been identified in mice as a distinct type of CD4^+^ T cells that express CD25 (Levings et al. 2002; Qiu et al. 2020; Sakaguchi et al. 2010). In the past decades, investigators have demonstrated that CD4^+^CD25^+^ Tregs play an important role in peripheral immunity and the prevention of autoimmunity (Komatsu et al. 2014). These cells can produce a variety of effective anti-inflammatory cytokines such as TGF-β and IL-10, thus exerting immunosuppressive effects on effector T cells (Chen et al. 2005; Hang et al. 2019; Rubtsov et al. 2008) Researchers also confirmed that Treg cell frequency was positively correlated with the viral DNA titer during Herpes simplex virus infection (Yu et al. 2018), suggesting that immune regulation is a key target for controlling viral infection.

Contagious ecthyma (*orf*) is a contagious, epitheliotropic zoonotic disease caused by *orf* virus (ORFV), which causes pustules on the skin, lips, nose, and oral mucosa of sheep and goats (Bergqvist et al. 2017; Haig and Mercer 1998; Lawan et al. 2021; Tobler et al. 2022). ORFV, likes Herpes simplex virus 1 (HSV-1), is a double-stranded DNA virus capable of causing recurrent infections (Abu Ghazaleh et al. 2023; Farooq and Shukla 2012; Zhu and Viejo-Borbolla 2021). Studies have shown that antibodies have little protection against ORFV, so it is important to study exactly which types of cells play a role in resisting reinfection (Bergqvist et al. 2017; Haig and McInnes 2002). Previous studies have shown that Treg cells induced by stress lead to HSV-1 reactivation from latency and suppress CD8^+^ T cell function and prompt HSV-1 reactivation (Yu et al. 2018). The hepatitis B virus, also a double-stranded DNA, induces IL-8 and promotes intrahepatic Treg accumulation (Li et al. 2019a; Zhang et al. 2021). However, studies on the relationship between Treg and viral diseases are mostly centered on human diseases (Komatsu et al. 2014), mainly because the lack of species-specific Treg mAbs in animals. Therefore, the development of Treg monoclonal antibodies is extremely important for elucidating the regulation of Treg cells in the immune system after viral or bacterial infection.

In this study, we prepared soluble proteins of CD25 and CD4 by the *E. coli* expression system and obtained fluorescent mAbs C11 and D12 which specifically recognize *Capra hircus* Treg cells. Both C11 and D12 can recognize the corresponding natural protein by immunofluorescence staining and flow cytometry, and the combination of mAbs with different fluoresces can detect the Treg cell expression level in the lip tissue of goats infected with ORFV. Our study provides an effective tool for studying the changes in the number and function of Treg cells during the pathogenesis and development of ORFV-infected goats. Furthermore, the mAbs can be used to study the changes in the number and function of CD4^+^T cells, Treg cells, and other Th cell subsets.

## Materials and methods

### Animals

Six female BALB/c mice were purchased from the laboratory animal center of the Air Force Medical University, Xi’an City, Shaanxi Province, China. All mice were maintained in specific pathogen-free conditions and treated following the guidelines of the Care and Use of Laboratory Animals of the Ministry of Health, China. All mice in this study were sacrificed by spine dislocation during the experiment. The goats used in this study were all from the farms near the NWAFU. Anesthetics were used at the time of sample collection, and the animals were treated until recovery after sample collection.

### Isolation and culture of peripheral blood mononuclear cells (PBMCs) of goats

Peripheral blood (10 mL) was taken aseptically from the jugular vein of healthy adult goats and anticoagulated with heparin. The anticoagulant blood was superimposed 1:1 with the lymphocyte separation solution and centrifuged at 2000 rpm for 20 min. Intermediate layer cells were washed twice with 5 mL PBS and added 1 mL of RPMI medium containing 10% FBS and 5 ug/mL ConA (Sigma-Aldrich). The cell concentration was adjusted to 5 × 10^6^/mL, and the cells were cultured for 24 h with 37 ℃, 5% CO_2_.

### Generation of two mAbs

Expression vector PET32a and PGX-6P-1 were preserved in our laboratory. Recombinant expression and affinity column purification of the extracellular region of goat CD4 and CD25 genes were conducted in *E. coli* (Gao et al. 2019; Yang et al. 2019). Two recombinant strains were successfully constructed using expression vectors CD4-PGX-6P-1 and CD25-PET32a, which were named as “rCD4” and “rCD25.” Six-week-old female BALB/c mice were immunized four times with purified recombinant proteins, and the procedures for immunization and cell fusion using PEG1500 (Sigma-Aldrich) were performed as previously described (Wagner et al. 2006). Hybridoma lines producing mAbs that only recognized the recombinant proteins but not the tag protein were screened by ELISA. Positive hybridoma cells that had been cloned three times were used to make ascites (Li et al. 2019b; Liu et al. 2022; Ma et al. 2019). The ascites of mice were purified by protein G agarose (Beyotime, Shanghai, China), labeled with a conjugation kit (Bioss, Beijing, China).

### SDS-PAGE and Western blot

Prokaryotic expressed rCD25 and rCD4 were mixed with loading buffer containing β-mercaptoethanol and denatured at 100 °C for 10 min. The denatured sample was run on a 5% polyacrylamide concentrate gel and 15% polyacrylamide separate gel under 70 V for 130 min. One gel was stained with Coomassie brilliant blue and the other one was used for Western blot. Then blot membranes were blocked with 5% skim milk powder for 2 h and washed four times with TBST, 6 × His-Tag, or 6 × GST-Tag antibody (Sangon, Shanghai, China) incubated overnight with a 1:4000 dilutions. Next, peroxidase-conjugated goat anti-mouse IgG antibody (Bioss, Beijing, China), diluted 1:5000, was incubated in the membranes for 2 h at 37 °C. Finally, the protein signals were visualized using the chemiluminescence reagent ECL and Image Lab software.

### Fluorescence conjugation

The concentration of the two mAbs was adjusted to 2 mg/mL using PBS. FITC and PE were, respectively, dissolved in dimethylsulfoxide (DMSO) to 20 mg/mL. Next, they were evenly mixed with the antibody at a concentration ratio of 8:1 and remained at repose for 8 h at 4 ℃, protected from light. Afterward, NH_4_Cl was added to the mixed solution at a final concentration of 50 mM and the reaction was stopped at 4 ℃ for 1.5 h.

### Flow cytometry

10^6^ PBMCs were harvested at 800 g for 5 min, then anti-FcR (CD16/32, BD Biosciences) was added to block cells for 10 min, followed incubation with the CD4 or CD25 fluorescent antibodies for 30 min. Afterward, cells were washed with PBS and centrifuged at 6000 rpm for 2 min at 4 ℃ and were analyzed by flow cytometry (BD FACSA, USA).

### Immunofluorescence assay

Frozen histological sections of goat lip (health and ORFV-infected) or thymus tissues were prepared. First, slides were fixed with antigen retrieval bufferson for 10 min, and then blocked with 5% donkey serum for 2 h. Next, slides were incubated overnight at 4 ℃ with CD4 or CD25 fluorescent antibody. Finally, using DAPI (BioFROXX, Guangzhou, China) to stain the nuclei, and images were captured using confocal laser scanning microscopy (Leica TCS SP8, Germany). It should be noted that between each two steps of this experiment, the slides were washed with PBS seven times for 10 min each time.

## Results

### Construction of recombinant expression plasmid of CD4 and CD25 genes in goats

Both CD4 (gene ID: 102170124) and CD25 (gene ID: 102170059) are transmembrane-spanning proteins that are very difficult to be soluble. Therefore, SignalP and TMHMM were used to analyze the structure of the two proteins (Fig. S1), and the extracellular region fragment of coding sequence was selected for gene cloning and vector construction (Fig. S2).

### Preparation of mAb against goat CD4

**Fig. 1:**
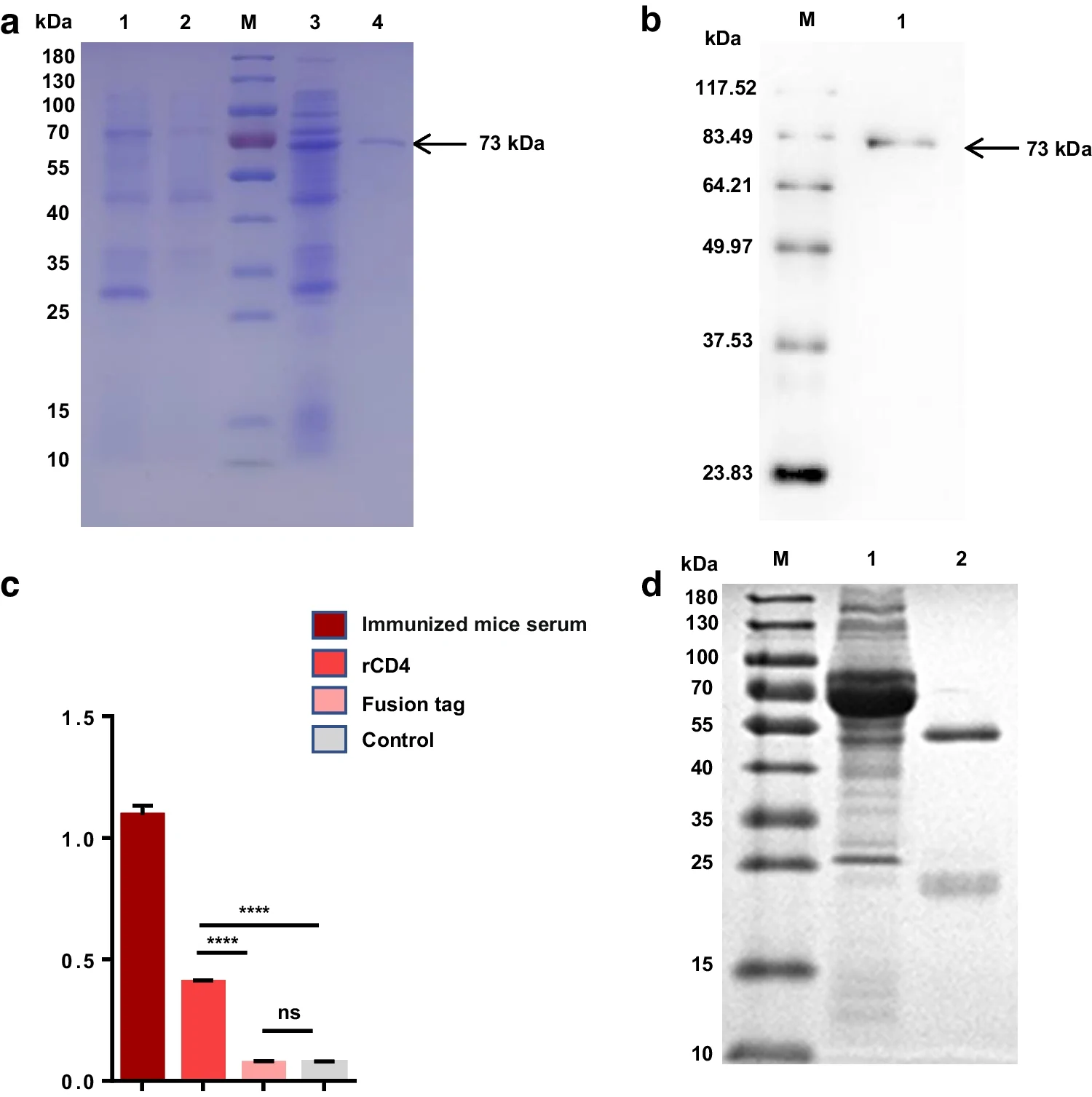
Preparation of mAb against goat CD4. **a** Expression, purification of caprine rCD4. M, marker; lane 1, induced PGEX-6P-1; lane 2, uninduced CD4-PGEX-6P-1; lane 3, induced CD4- PGEX-6P-1; lane4, purified rCD4 of 73 KDa. **b** Western blot analysis of purified rCD4. M, protein marker; lane 1, rCD4 protein. **c** Generation and specificity determination of hybridoma cells D12 by ELISA. purified rCD4 protein (1 μg), fusion tag protein of PGEX-6P-1 (1 μg) and PBST were used as antigens. The culture supernatant of D12 hybridoma cells was served as the primary antibody. Serum from immunized mice that reacted with both rCD4 and fusion tag protein served as positive controls (*n* = 3, ****P < 0.0001). **d** Western-blot analysis of the purification of mAb D12; M, marker; lane 1, unpurified BALB/c ascites fluid; lane 2, purified BABL/C ascites fluid

The recombinant plasmid rCD4 was transformed into the *E. coli* strain BL21 (DE3) and cultured in LB medium with Amp. In the indicated strains, OD600 was grown to 0.6, IPTG was added to induce protein expression, and the expressed protein was purified with GST-tag purification resin. SDS-PAGE analysis showed that the purified protein band was single, with a size of about 73 kDa (Fig. 1a), and the purified protein could react with GST antibody. The results showed that we obtained a single band of recombinant fusion protein suitable for the preparation of mAb (Fig. 1b). Purified recombinant CD4 protein was used as an immunogen and antigen to produce goat CD4 mAb. ELISA results showed that we screened a hybridoma cell line that positively reacted with recombinant protein, but not the tag protein (Fig. 1c). The screened positive hybridoma cells were cultured and expanded, then they were injected into the abdominal cavity of BALB/c mice to prepare ascites, and protein G agarose was used to purify the ascites. SDS-PAGE analysis showed that we successfully prepared CD4 monoclonal antibody and named it D12 (Fig. 1d).

### Preparation of mAb against goat CD25

**Fig. 2:**
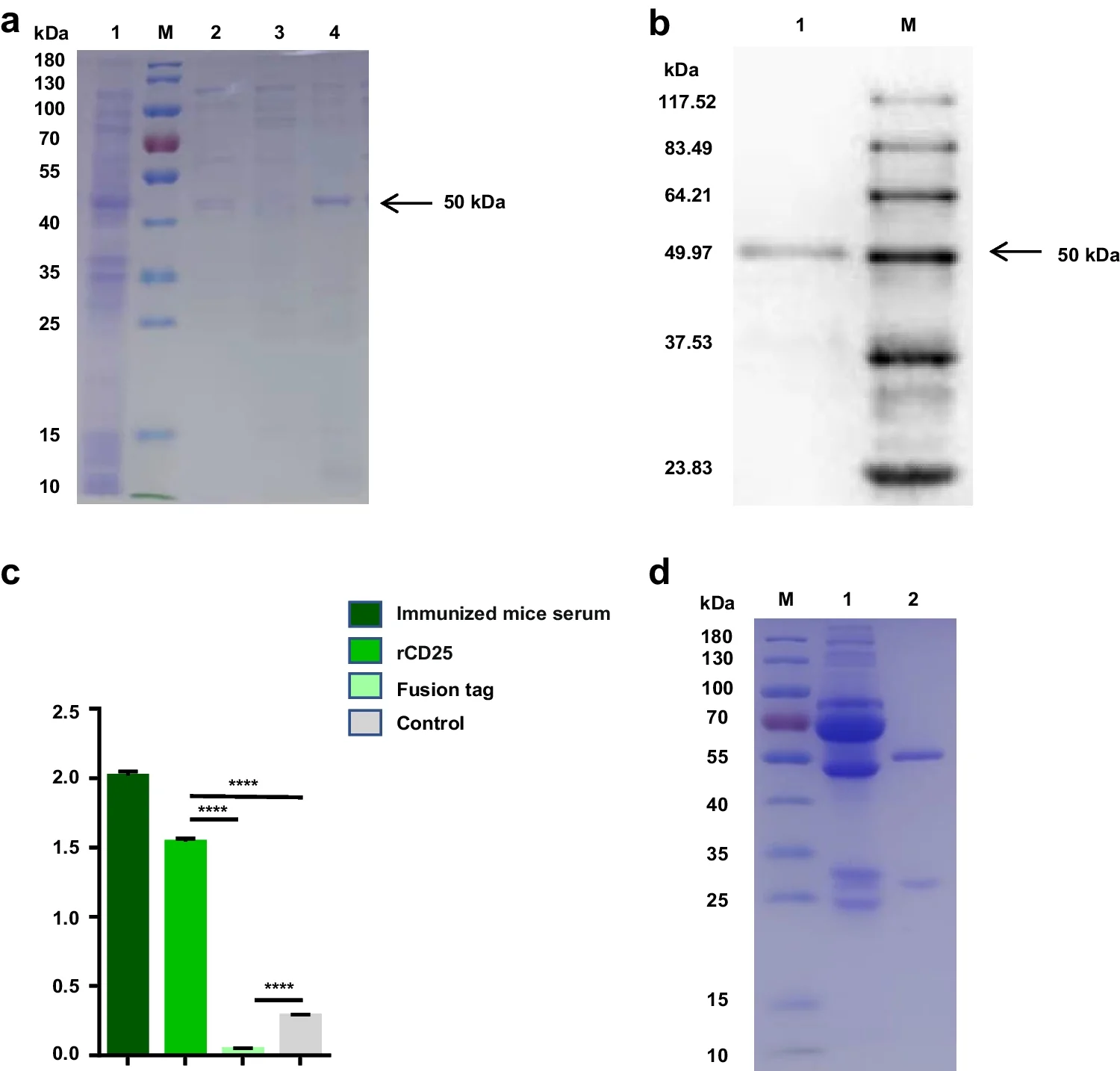
Preparation of mAb against goat IL-2RA. **a** Expression, purification of caprine rIL-2RA-PET32a. M, marker; lane 1, induced rIL-2RA-PET32a; lane 2, eluents for the unpurified supernatant flowed through the column; lanes 3–4, eluents after the column was washed with 20 mM and 100 mM imidazole, respectively. **b** Western blot analysis of reactivity of protein rIL-2RA. M, protein marker; lane 1, rIL-2RA protein. **c** Generation and specificity determination of hybridoma cells D12 by ELISA. Purified rCD4 protein (1 μg), fusion tag protein of PET-32a (1 μg), and PBST were used as antigens. The culture supernatant of C11 hybridoma cells was served as the primary antibody. Serum from immunized mice that reacted with both rCD4 and fusion tag protein served as positive controls (*n* = 3, ****P < 0.0001). **d** Western-blot analysis of the purification of mAb C11; M, marker; lane 1, unpurified BABL/c ascites fluid; lane 2, purified BABL/c ascites fluid

The *E. coli* strain Trans B (DE3) that contained recombinant plasmid rCD25 was cultured in LB medium and IPTG was added to induce its expression. After induction, the recombinant fusion protein CD25 was purified with Ni–NTA His bind resin, and the purified protein was analyzed by SDS-PAGE and Western blot. The results showed that we obtained a single protein band about 50 kDa (Fig. 2a) and it could bind to 6X His-Tag monoclonal antibody (Fig. 2b). These results suggest that the recombinant fusion protein rCD25 is suitable as an immunogen. The purified recombinant protein rCD25 was used to immunize BALB/c mice for four times, then spleen cells derived from BALB/c mice were fused with SP2/0 cells. After 9 days, hybridoma cells reacted with the recombinant protein but not the fusion tag were screened (Fig. 2c). Positive hybridoma cells were expanded culture to produce ascites, which were purified by protein G agarose. The results showed that we successfully prepared CD25 monoclonal antibody and named it C11 (Fig. 2d).

### Characteristics of mAb D12 and C11

**Fig. 3:**
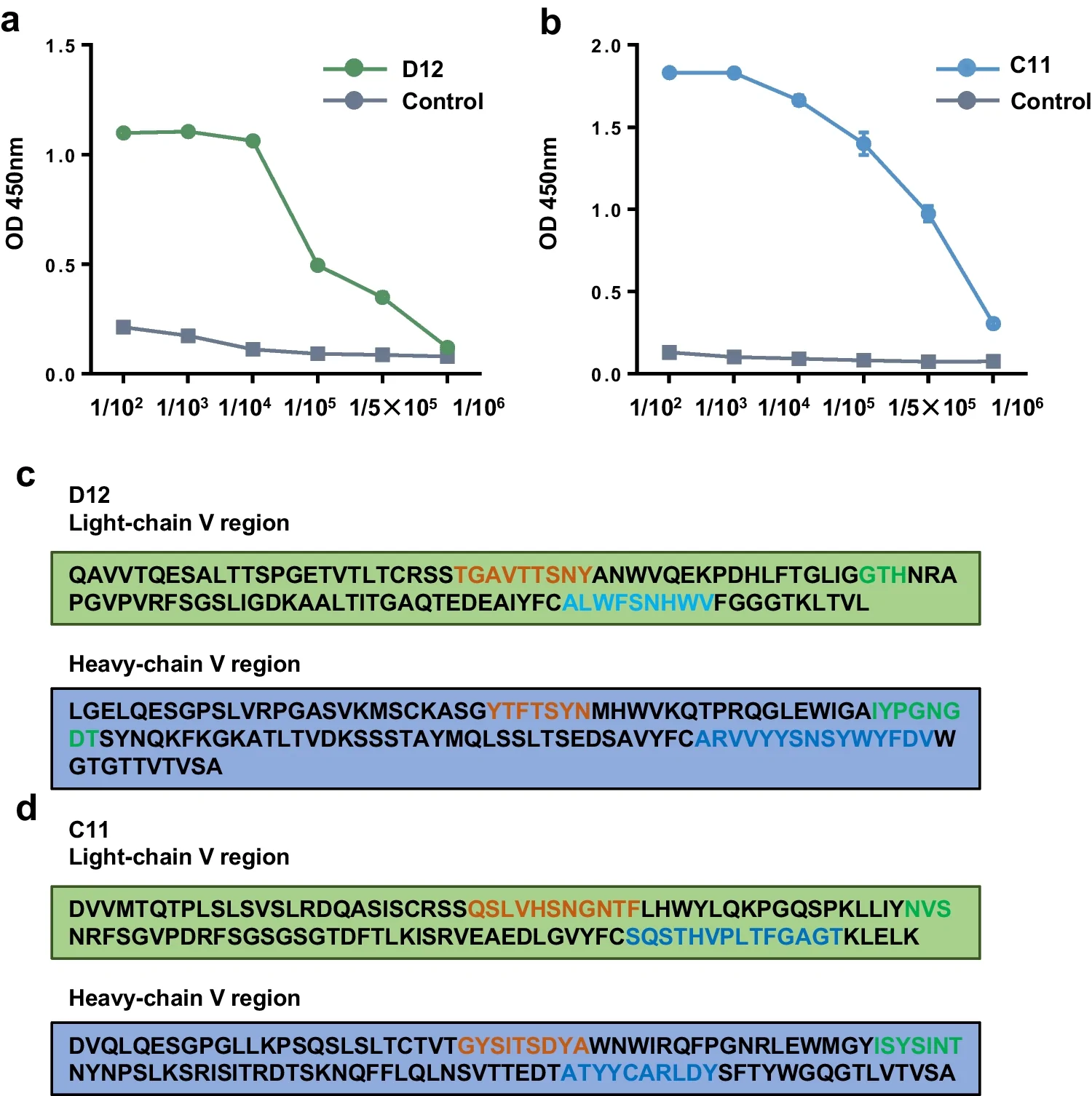
Characteristics of mAb D12 and C11. **a** The affinity of mAb D12 was determined by ELISA at different dilutions. **b** The affinity of mAb C11 was determined by ELISA at different dilutions. **c**, **d** The sequences of D12 and C11 were analyzed using Vbase2 and IMGT/V Ques (tawny, CDR1; green, CDR2; blue, CDR3)

To confirm the affinity of the monoclonal antibody, ELISA was used to detect the reaction of the purified antibody with the corresponding recombinant protein at different dilutions, and the results showed that both antibodies had high titer (1:500,000), which meant that they had good affinity (Fig. 3a, b). The sequence diversity of the complementarity determining region (CDR) determines the specificity and affinity of antibodies, because they recognize and bind to specific epitopes by interacting with antigens, so we detected the CDR sequences of two monoclonal antibodies (Fig. 3c, d), which laid a foundation for further understanding and validation of the molecular mechanism of “CDRs-epitope”-specific binding.

### Identification of two monoclonal antibodies specific for Treg cells of goat

**Fig. 4:**
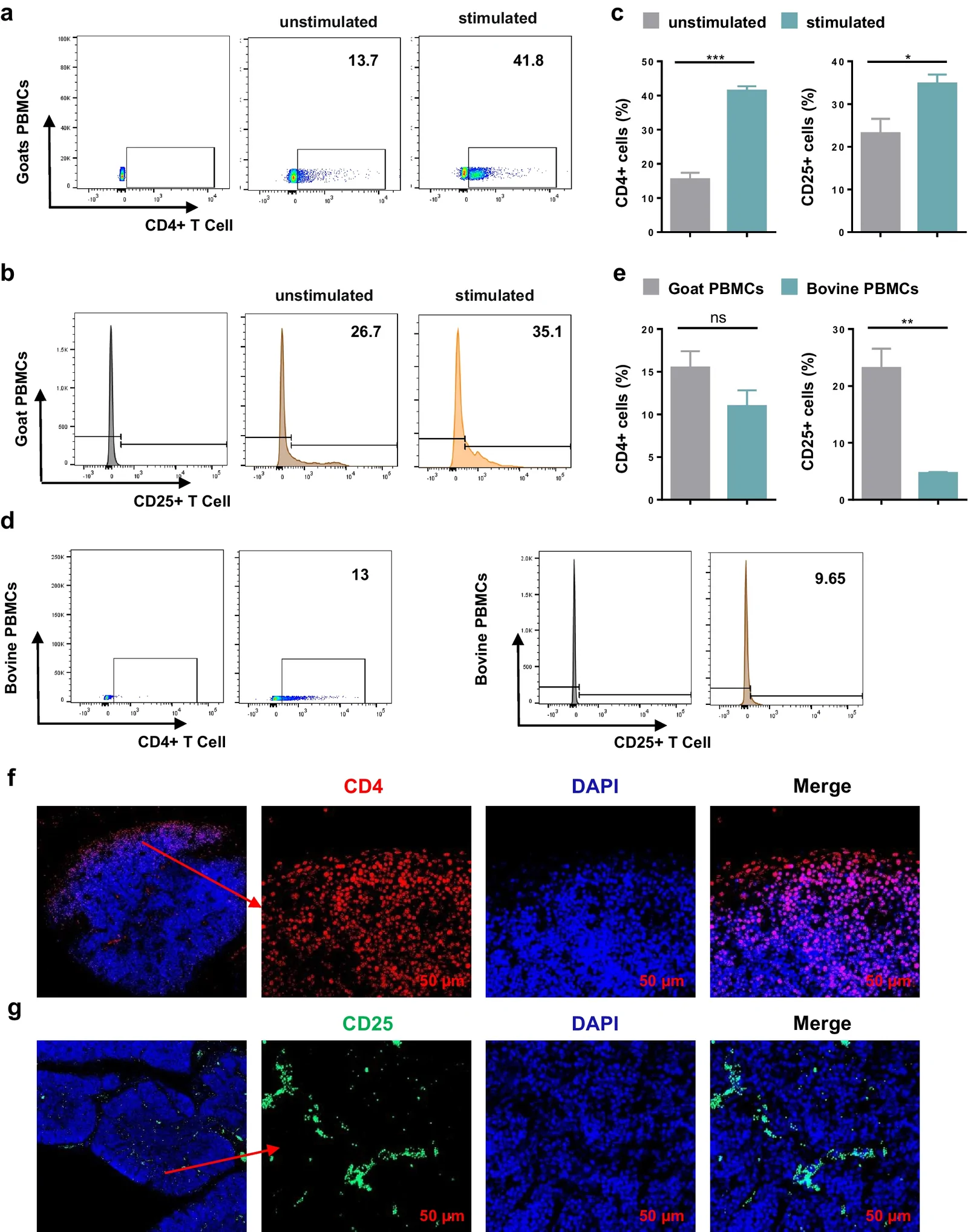
Application of the mAb D12 and C11 in flow cytometry and immunofluorescence staining. **a**, **b**, **d** Representative FACS plots. **c** Significance difference analyzes the expression of CD4^+^ cells and CD25 + cells in unstimulated or Con A-stimulated goat PBMCs (*n* = 3). **e** Significance difference analyzes the expression of CD25^+^ cells and CD4.^+^ cells in bovine and goat PBMCs by using mAb C11 and mAb D12 (*n* = 3). **f** Immunofluorescence staining of goat thymus using mAb D12 (*n* = 3). **g** Immunofluorescence staining of goat thymus using mAb C11 (*n* = 3)

To detect whether these two antibodies could recognize natural goat protein, the expressions of CD4 and CD25 were detected by flow cytometry, which in healthy goat’s PBMCs cultured for 24 h with or without 5 μg/mL ConA. Finally, we found that ConA-stimulated PBMC cells expressed more CD4 and CD25 production than unstimulated PBMC cells, demonstrating that antibodies can be applied to flow cytometry (Fig. 4a–c). To assess the specificity of the antibody, PBMC cells from peripheral blood of healthy cattle were isolated and analyzed by flow cytometry. The results showed that goat CD4 antibody also recognized bovine CD4 molecule, but the affinity of CD25 antibody and bovine surface CD25 molecule was not high, indicating that CD25 antibody was more specific to goats (Fig. 4d, e). As we all know, two main histological regions of the thymus, the cortex and the medulla, are separated by a border zone named the cortico-medulla junction (Bódi et al. 2019; Gordon and Manley 2011; Gulla et al. 2023; Sebastianelli et al. 2020); CD4/CD8 double-positive thymocytes are present in the thymic cortex and have large numbers. In addition, T cells expressing CD25 are also abundant in the thymus. To further demonstrate that our prepared mAbs can recognize natural cells, we analyzed the expression of CD4 and CD25 in thymus tissue of 1-month-old goats by immunofluorescence analysis. The results showed that the number of CD4^+^ T cells in the outer cortical area, the inter cortical area, and the medulla area decreased gradually (Fig. 4f). The thymus cortex forms arcuate postcapillary venules at the cortex-medulla junction, which are the main channels for thymus cell output. The staining results showed that CD25^+^ cells formed arcuate staining at the cortex-medulla junction (Fig. 4g). These results indicated that both antibodies can recognize natural CD4 and CD25 proteins. Moreover, it can be used in flow cytometry and immunofluorescence staining.

### Application of the mAb D12 and C11 in contagious ecthyma caused by orf virus

**Fig. 5:**
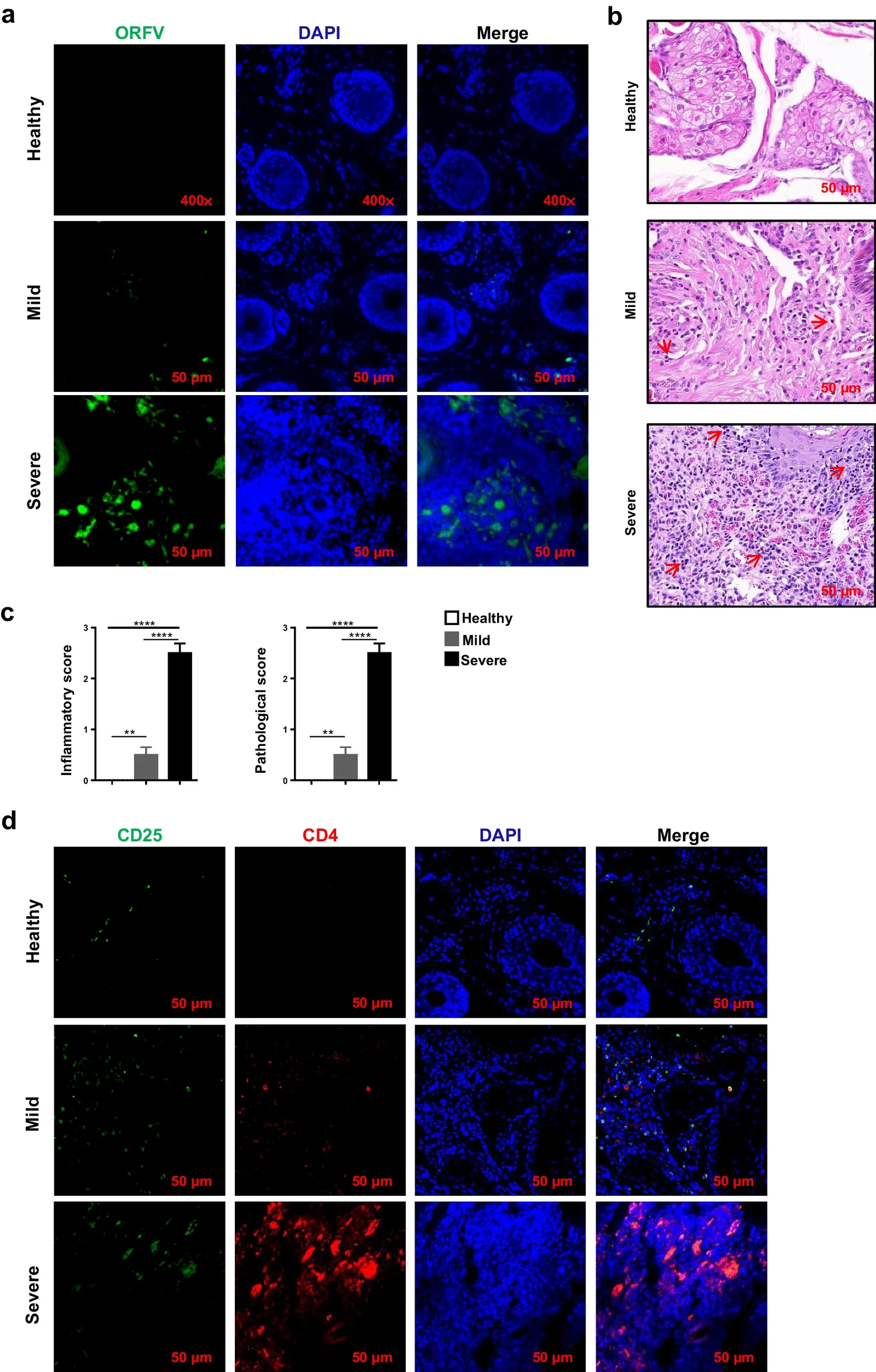
Application of the mAb D12 and C11 in contagious ecthyma. **a** Non-infected or ORFV-infected goat (mild or severe) lip tissues as analyzed by immunofluorescence staining using ORFV mAb. **b** Non-infected or ORFV-infected goat (mild or severe) lip tissues as analyzed by HE staining. **c** Inflammatory score and pathological score of non-infected or ORFV-infected goat (mild or severe) lip tissues. **d** Non-infected or ORFV-infected goat (mild or severe) lip tissues as analyzed by immunofluorescence staining using mAb D12 and C11

In general, the same-species Fc fragments have the same structure. To avoid non-specific binding of Fc fragments with fluorescent secondary antibodies, we used fluorescent conjugation kit to conjugate mAb D12 with PE dye and mAb C11 with FITC dye. To demonstrate the role of Treg cells in ORFV invasion of the organism, we selected goats at different stages after ORFV infection. PCR results of specific primers of virulence gene B2L of ORFV showed that the virus was indeed present in the lip tissue of infected goats (Fig. S3). IFA and HE results showed that the viral load and inflammatory response in the tissue of goats in mild group were lower than those in severe group (Fig. 5a–c). Meanwhile, the presence of CD4^+^CD25^+^ Treg cells in the lip tissue of goats at different stages after infection was detected by fluorescently coupled antibodies. The results showed that the number of CD4^+^ T cells, CD25^+^ cells, and CD4^+^CD25^+^ Treg cells in the lip tissue of severe group was significantly higher than that of mild group (Fig. 5d). This suggests that the increase may be positively related to the inflammatory response, perhaps implying that the virus affects the inhibitory function of Tregs.

## Discussion

Currently, the species-specific mAbs of Treg studies in goats are still not available; the role of Treg cells in contagious ecthyma have not been studied. For now, the expression level of Treg is monitored by measuring the mRNA expression of FOXP3, which is the Treg transcription factor. However, the transcript level did not coincide with the protein expression levels of the gene (Liu et al. 2016). Therefore, real-time quantitative PCR is often combined with flow cytometry to further detect the number of Treg cells. When the intracellular antigen was detected by flow cytometry, the cells were fixated and ruptured off the membrane, then the mAbs were added. Membrane-breaking agents created pores in the flowing and intact cell membrane to facilitate antibody entry, but it also caused cell death, and we could not collect viable cells for functional verification experiments. To avoid this problem, we picked the membrane proteins CD4 and CD25 to prepare mAbs to obtain viable Treg cells, which can be used to understand the modification of function and differentiation of Treg cells after virus infection goats (Jansen et al. 2021; Yu et al. 2020).

CD25 and CD4 are considered phenotypic markers on the surface of regulatory T cells (Cheung et al. 2022; Ng et al. 2001). In this study, our data demonstrate that in local lesion tissues, Treg cells are recruited to the site of inflammation in ORFV-infected goats (Fig. 4c, d). We also confirmed that the Treg cell frequency was positively correlated with the virus concentration in tissues during ORFV infection (Fig. 4b–d). Numerous studies showed that the inflammatory environment can cause downregulation of the inhibitory ability of Treg cells and even endow the cells with pro-inflammatory properties (Izcue et al. 2008; Oldenhove et al. 2009; Thomas et al. 2018). A large number of inflammatory cells infiltrated the lip tissue of goats infected with *orf* virus (Fig. 4 b, c). Therefore, we suspect that treg regulation is inhibited in ORFV-infected goat lip tissue, resulting in excessive tissue damage, which needs further experimental studies to explore.

In this study, BALB/c mice were immunized with CD4 and CD25 recombinant fusion proteins expressed in prokaryotes, and the hybridoma cell lines that indirectly specifically recognized Treg cells of goat were screened by ELISA. At the same time, to verify whether the staining results of D12 and C11 in thymus tissue are non-specific, and to exclude the phenomenon of spontaneous fluorescence in thymus tissue (Sainte-Marie 1966), we used homologous control antibodies. The results showed that the homologous control antibody could not stain the thymus tissue (Fig. S4), which proved that the antibody could be applied to IFA. In addition, flow cytometry was used to analyze the expression of CD4 and CD25 in PBMC of goats and cattle. The results showed that mAb C11 had good species specificity (Fig. 3c); mAb D12 can be used in goats and cattle. Meanwhile, the fluorescent CD4 antibody conjugate can be used in combination with IL-17, IFN-γ, and other antibodies to detect the changes of Th1, Th17, and other Th cells during virus infection (Fig. S5), providing scientific support for further research into the role of helper T cells in ORFV-infected animals.

In conclusion, the hybridoma cell lines C11 and D12 were able to recognize natural CD4 and CD25 proteins, but not fusion tag proteins, with excellent specificity. These mAbs can be applied to flow cytometry and immunofluorescence staining. Our results indicate an important role of Treg cells during *orf* virus infection and suggest a potential tool to accurately detect goat Treg cells. In addition, these mAbs could provide strong support for the study of long-term cellular immunity against reinfection in nonlymphoid tissues.

## Supplementary Information

Below is the link to the electronic supplementary material.

Supplementary file1 (PDF 731 KB)

## Data Availability

The data presented in this study are available on request from the corresponding author Denkun Chen.
