# Supplementary material for: Preparation and application of fluorescent monoclonal antibodies recognizing goat CD4+CD25+ regulatory T cells
Source: Appl Microbiol Biotechnol. 2024 May 8;108:327. doi: 10.1007/s00253-024-13115-4 (PMC11078799; doi:10.1007/s00253-024-13115-4)
Supplement: Supplementary file 1 [file 253_2024_13115_MOESM1_ESM.pdf]

Supplementary Information

Applied Microbiology and Biotechnology

Title

Preparation and application of fluorescent monoclonal antibodies recognizing goat $CD4^{+}CD25^{+}$ regulatory T cells

Authors

Yunpeng Wang[a,1], Haoyue Yang[a,1], Jiajin Hu[1], Yuecai Jiang[1], Wentao Ma[1]*, Shikong Gao[2]*, Dekun Chen[1]*

[a] Co-author

[1] College of Veterinary Medicine, Northwest A&F University, Yangling, Shaanxi, China

[2] Shenmu Animal Husbandry Development Center, Shenmu 719300, Shaanxi, China

* Co-corresponding author

Co-corresponding author

E-mail:mawentao@nwafu.edu.cn (WT.M.), gsk20131001@163.com (SK.G.), or cdk@nwafu.edu.cn (DK.C.).

A

1 ATGGGCCCGGGGACCTCTCTTAGGCACCTGTTCTTGGTGCTGCAACTGGTGATGCTCCCG
1 **M G P G T S L R H L F L V L Q L V M L P**

61 GCTGGCACTCAGGGAAAAGCAGTGGTGCTGGGTAAGGCAGGAGGCCAGGCAGAGCTGCCC
21 **A G T Q G** K A V V L G K A G G Q A E L P

121 TGCCAGGCTTCCCAGAAGAAGAACATAGTCTTCAGCTGGAAAGATTCTTCCCAGTCCAAG
41 C Q A S Q K K N I V F S W K D S S Q S K

181 ATTCTGGGGAGTCATAACTCCTTCTTGCACAAAGGTAACACTGAACTGAGCCGTCGAGTG
61 I L G S H N S F L H K G N T E L S R R V

241 GAATCCAAAAGAAACCTGTGGGACCAAGGATCCTTTCCTCTGATCATCAAGAACCTCCAA
81 E S K R N L W D Q G S F P L I I K N L Q

301 GTAACTGACTCGGGGACTTATACCTGTGAAGTGGATAGCAAGAAGCTCGAGGTGGAACTG
101 V T D S G T Y T C E V D S K K L E V E L

361 AAGGTGTTCGGACTGACTGCCAGCTCGGACACCCGTCTACTGCTGGGACAGAGCCTGACC
121 K V F G L T A S S D T R L L L G Q S L T

421 TTGACTTTGGAGAGTCCCTCTGGGAGCAACCCTTCTGTGCAGTGGAAGGGTCCAGGGAAT
141 L T L E S P S G S N P S V Q W K G P G N

481 AATAGGAAGGAAGAACTCAAGAGCCTGTCACTGGCCCAGGTGGGGCTGCAGGACAGTGGT
161 N R K E E L K S L S L A Q V G L Q D S G

541 ACCTGGACGTGCACTATCTCCCAGAGCCAGCAGACACTGGAGATCAAAATACCCATCGTG
181 T W T C T I S Q S Q Q T L E I K I P I V

601 GTGCTGGCCTTCCAGAAGGCCCCCGAAACAGTCTATGTGAAGGAGGGGGAGCAGGCGGAG
201 V L A F Q K A P E T V Y V K E G E Q A E

661 TTTTCCTTCCCACTCACCTTCGAGGATGAAAATCTGAGTGGGGAGCTGACTTGGCAGCAG
221 F S F P L T F E D E N L S G E L T W Q Q

721 GCGAACAAGGATTCTTCCTCCCAATCCTGGGTCACCTTCACCCTGAGGAACAGAGAGGTG
241 A N K D S S S Q S W V T F T L R N R E V

781 AAGGTGAACAAGACTCACAAGGACCTCAAGCTCCGTGTGGAGGAGAGGCTACCGCTGCGT
261 K V N K T H K D L K L R V E E R L P L R

841 CTCACTCTGCTGCGGACCTTGCCTCAGTACGCGGGTTCTGGAACCCTGACCCTGGATCTC
281 L T L L R T L P Q Y A G S G T L T L D L

901 TCCAAGGGGAAGCTGCATCAGAAAGTGAACCTCGTGGTGATGAGAGTGACTAAGTCCCCA
301 S K G K L H Q K V N L V V M R V T K S P

961 AACAGTCTGACCTGTGAGGTGCTGGGGCCCAGCCCCCCAAGGCTGACCCTGAACTTGAAG
321 N S L T C E V L G P S P P R L T L N L K

1021 CTGGGGAACCAGAGTATGAAGAGCTCAAATCAGCCAAAGTTGGTGACAGAGCCGGAACCC
341 L G N Q S M K S S N Q P K L V T E P E P

1081 AAGGCTGGGATGTGGCAGTGTCTGCTGAGTGACCAGGGCAAAGTCCTGCTGGAATCCAAG

361 K A G M W Q C L L S D Q G K V L L E S K

1141 ATCGAGGTCTTGCCGTCAGAGTTCATCCAGGCCTGGCCGATGCTCCTGCCCATGGTGTTG
381 **I E V L P S E F I Q A W P M L L P M V L**

1201 GGGGGAATCGCAGGCCTAGCGCTTCTCACTGGCTCCTGCATCTTCTGTGTTAAATGCTGG
401 **G G I A G L A L L T G S C I F C V K C W**

1261 CACCGCAGGCGCCAGGCAGAACGGATGTCTCAAATCAAGAGGCTCCTCAGTGAGAAGAAG
421 **H R R R Q A E R M S Q I K R L L S E K K**

1321 ACCTGCCAGTGCCCCCACCGTCTCCAGAAGACCCACAGTCTCACCTGA
441 **T C Q C P H R L Q K T H S L T ***

B

1 ATGTCGGCAGTGGTCCCAGGGGCCAGGAGGATGGAGCCAAGCTTGCTGATGTGGAGGTTC
1 **M S A V V P G A R R M E P S L L M W R F**

61 TTCGTATTCATCGTGGTACCTGGCTGCGTGACAGAGGCTTGTTATGATGACCCTCCGAGA
21 **F V F I V V P G C V T E A** C Y D D P P R

121 CTCAGAAACGCCATGTTCAAGGCCCTCAGGTACGAGGTGGGCACCATGATAAACTGCGAC
41 L R N A M F K A L R Y E V G T M I N C D

181 TGCAAGGCCGGCTTCCGCAGGGTGTCGGCCGTCATGCGCTGCGTGGGGGACTCCAGCCAC
61 C K A G F R R V S A V M R C V G D S S H

241 TCTGCCTGGAACAACAGATGCTTCTGCAACAGCACCTCCCCTGCTAAGAACCCAGTAAAA
81 S A W N N R C F C N S T S P A K N P V K

301 CCAGTTACTCCTGGATCCGAAGAACAGAGGGAGAGAAAACCCACAGATGCACAGAGCCAA
101 P V T P G S E E Q R E R K P T D A Q S Q

361 ACGCAGCCTCCGGAGCAAGCTGACCTTCCAGGTCACTGCGAGGAACCACCGCCATGGGAA
121 T Q P P E Q A D L P G H C E E P P P W E

421 CATGAACGTGAACCTTTGAAGAGAGTCTATCATTTCACGCTGGGGCAGACGGTTCACTAC
141 H E R E P L K R V Y H F T L G Q T V H Y

481 CAGTGTGCCCAGGGATTCAGGGCCCTACACACCGGTCCTGCTGAAAGCACCTGCACGATT
161 Q C A Q G F R A L H T G P A E S T C T I

541 ATCCACGGGGAGATGAGGTGGACCAGGCCCAGGCTCAAGTGCATAAGTGAAGGGGCGAAC
181 I H G E M R W T R P R L K C I S E G A N

601 AGTCAGGCTCCAGATGAAGCAGAGCCTCCGGAGAGCACGGAAGCTCCACCTGGGAGTGGA
201 S Q A P D E A E P P E S T E A P P G S G

```
661    ACTTTCTTAACAACCAGGACGGCAGGGACCACAGATTTCCAGAAGCCCACACGCGTGGTT
221     T  F L  T T R  T A  G T  T D F  Q K  P T R  V V

721    GCAACGCTGGATACGTTCATATTTACCACTGAGTACCAGATCGCAGTGGCCGGCTGCATC
241     A  T L  D T F  I F  T T  E Y Q  I A  V A G  C I

781    CTCCTGCTCTCCAGCGTCCTCCTCCTGAGCTGCCTCACGTGGCAGCGGAGATGGAAGAAG
261     L  L L  S S V  L L  L S  C L T  W Q  R R W  K K

841    AACAGAAGGACAATCTAG
281     N  R R  T I *
```

Supplemental figure S1.

Structural analysis of CD4 and CD25 genes in goats.

A, CD4 coding sequence region. B, CD25 coding sequence region. Signal peptide (red), extracellular region (black), transmembrane region (purple), intracellular region (green).

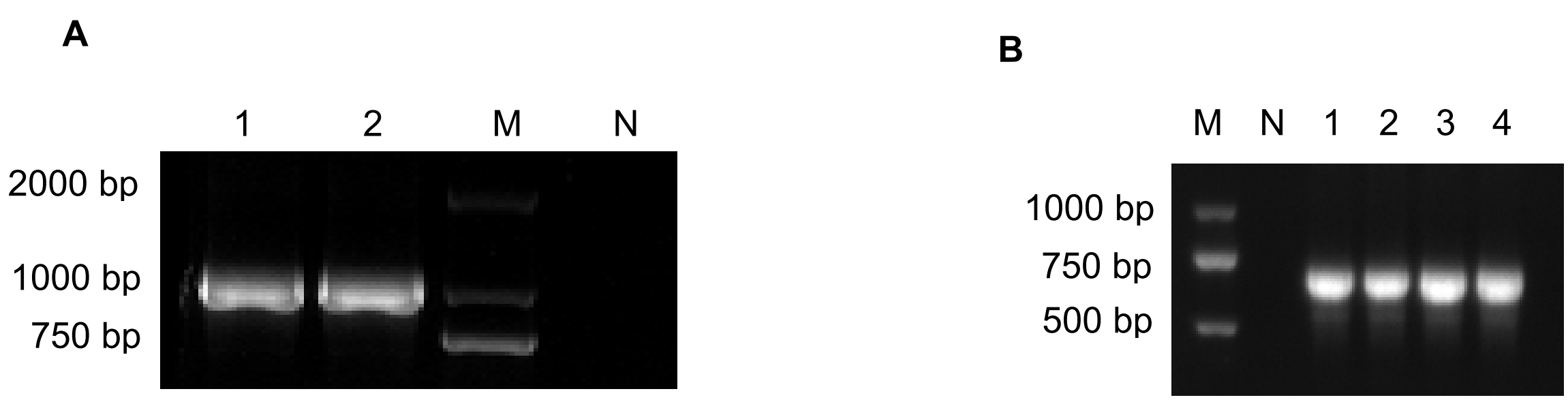

Supplemental figure S2.

Colony PCR analysis of the recombinant CD4 and CD25 plasmid.

A, Pcr results of recombinant plasmid CD4-PGX-6P-1. B, Pcr results of recombinant plasmid CD25-PET32a. M, DL2000 DNA marker. N, Negative controls. Lane 1-2, two different monoclonal colonies(A). Lane 1-4, four different monoclonal colonies(B).

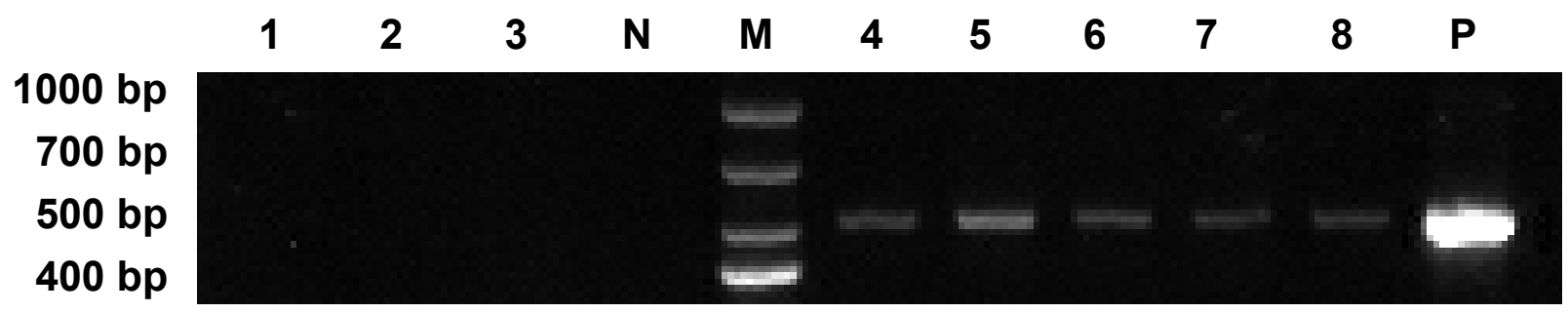

Supplemental figure S3.

Analyze B2L gene of Orf virus using samples of goat lip tissues.

M, DL2000 Maker; N, negative control; P, positive control; lane 1-3, healthy goat lip tissues; lane4-8, Orf virus-infected goat lip tissues.

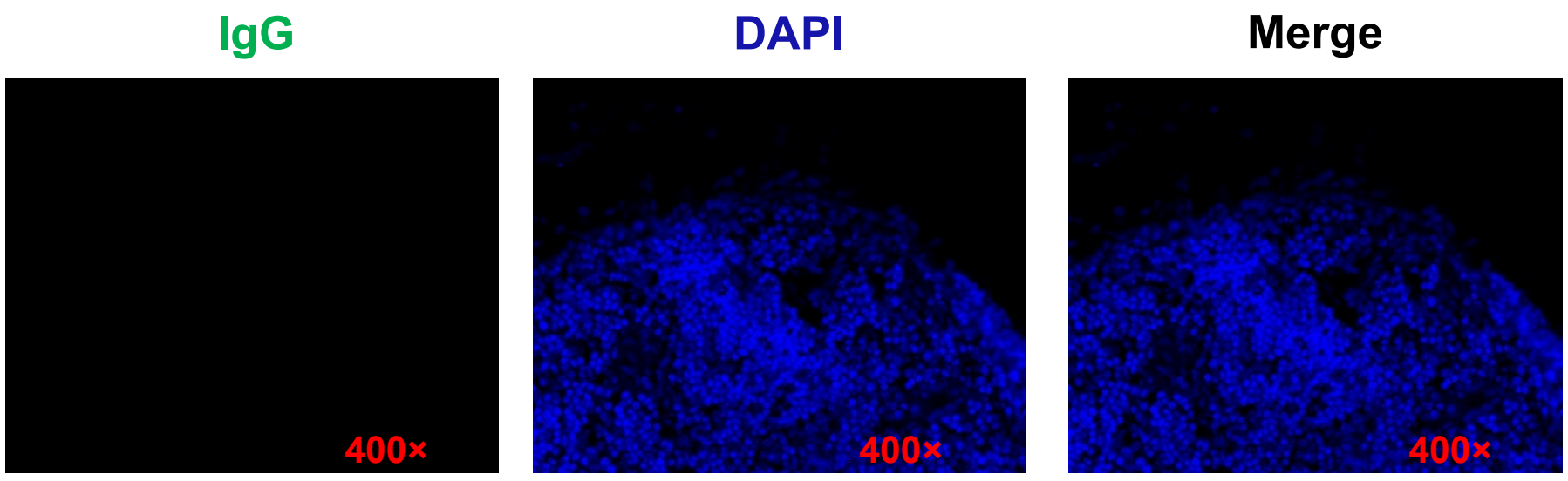

Supplemental figure S4.

Results of immunofluorescence homologous control antibody staining of goat thymus tissue. Homologous control antibodies do not stain.

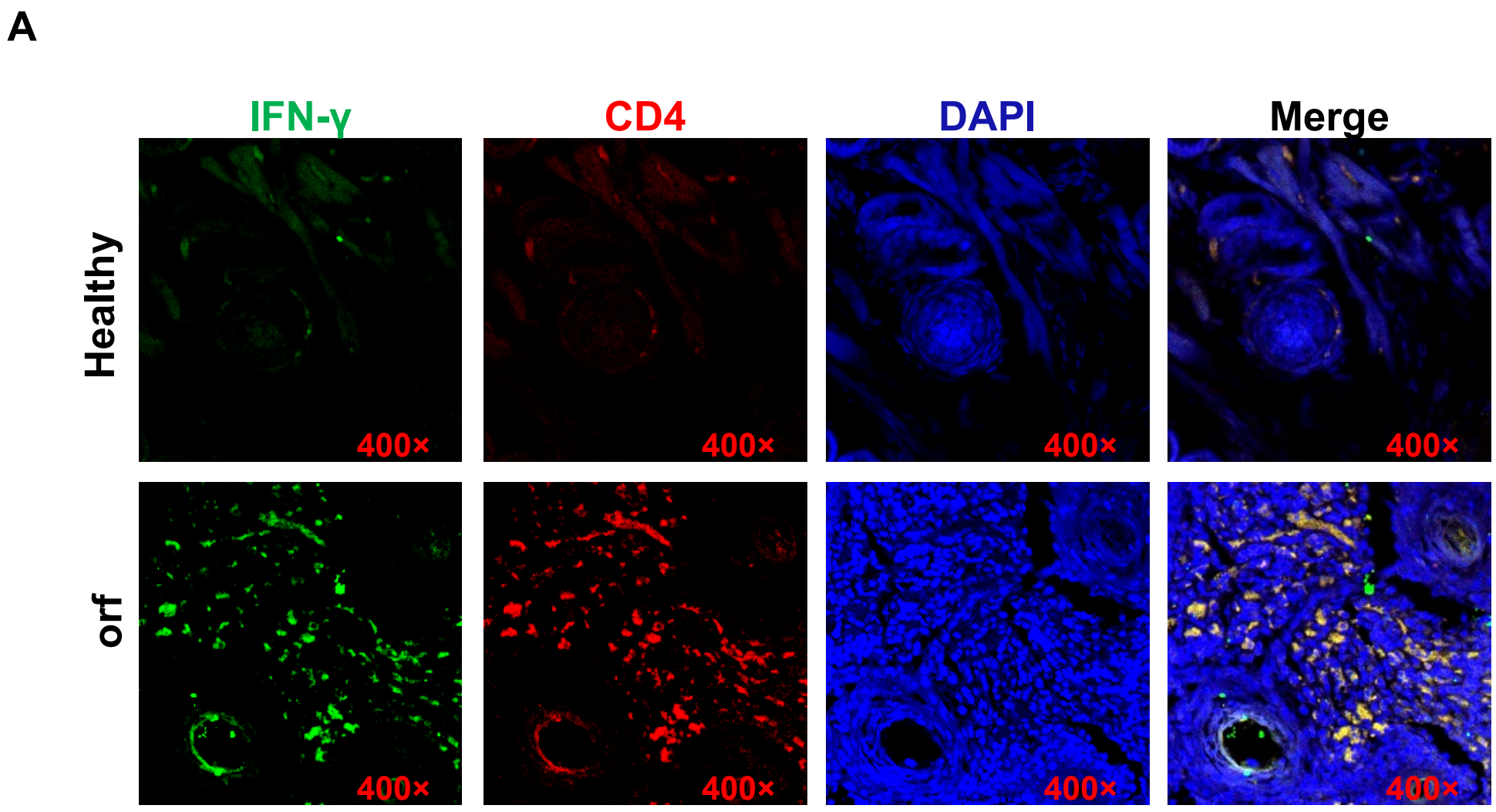

B

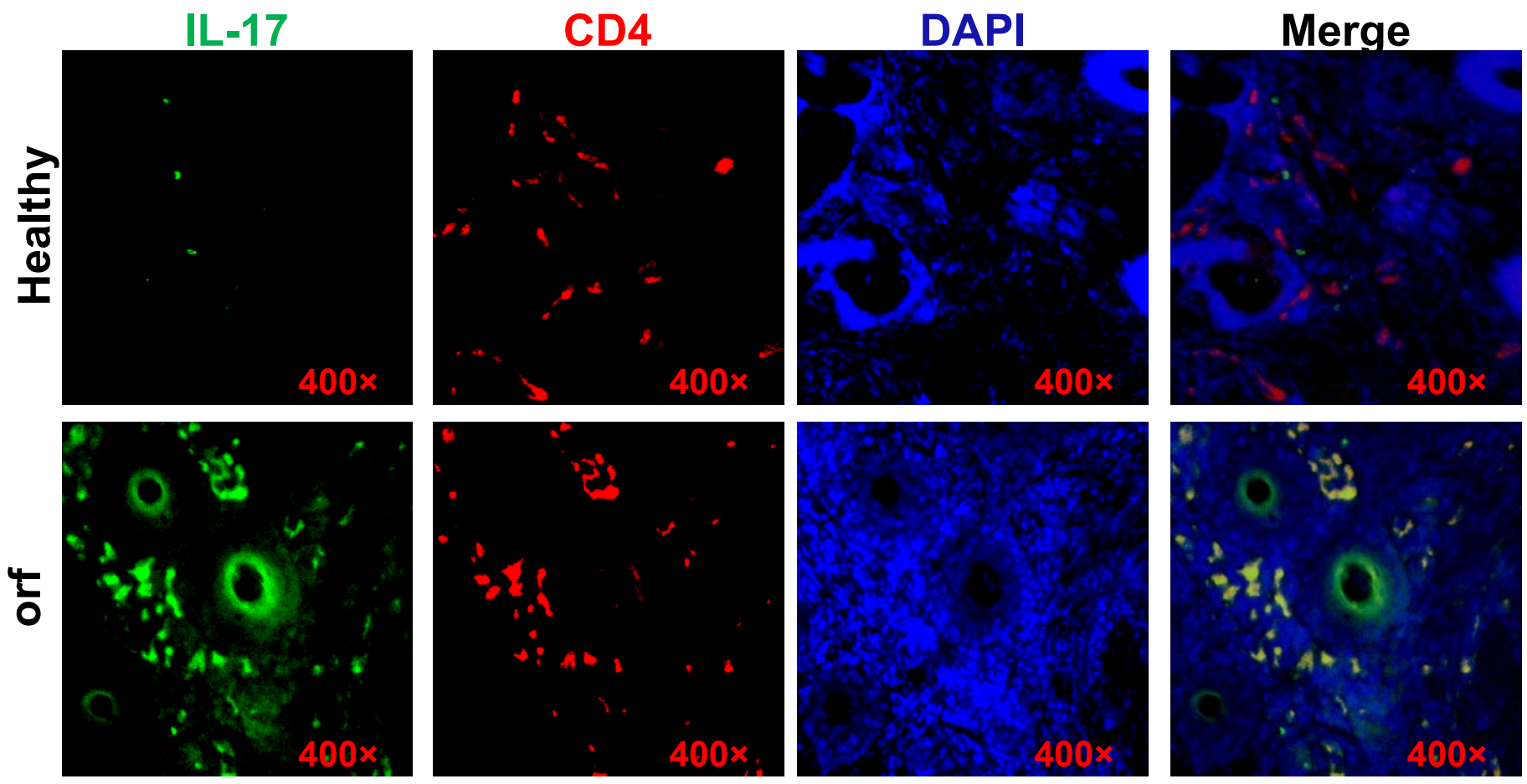

Supplemental figure S5.

Detection of Th1 and Th17 cells in goat lip tissue

A, Immunofluorescence staining of the lip tissues in healthy and Orf virus-infected goat using mAb D12 and IFN-γ mAb, Nuclear staining with DAPI. Th1 cells accumulated in the lip tissue of Orf virus-infected goat.

B, Immunofluorescence staining of the lip tissues in healthy and Orf virus-infected goat using mAb D12 and IL17 mAb, Nuclear staining with DAPI. Th17 cells accumulated in the lip tissue of Orf virus-infected goat.
