# Supplementary material for: Preparation and application of fluorescent monoclonal antibodies recognizing goat CD4+CD25+ regulatory T cells
Source: Appl Microbiol Biotechnol. 2024 May 8;108(1):327. doi: 10.1007/s00253-024-13115-4 (PMC11078799; doi:10.1007/s00253-024-13115-4)
Supplement: Supplementary file 1 — Supplementary file1 (PDF 731 KB) [file 253_2024_13115_MOESM1_ESM.pdf]

Supplementary Information

Applied Microbiology and Biotechnology

Title

Preparation and application of fluorescent monoclonal antibodies recognizing goat $CD4^{+}CD25^{+}$ regulatory T cells

Authors

Yunpeng Wang[a,1], Haoyue Yang[a,1], Jiajin Hu[1], Yuecai Jiang[1], Wentao Ma[1]*, Shikong Gao[2]*, Dekun Chen[1]*

[a] Co-author

[1] College of Veterinary Medicine, Northwest A&F University, Yangling, Shaanxi, China

[2] Shenmu Animal Husbandry Development Center, Shenmu 719300, Shaanxi, China

* Co-corresponding author

Co-corresponding author

E-mail:mawentao@nwafu.edu.cn (WT.M.), gsk20131001@163.com (SK.G.), or cdk@nwafu.edu.cn (DK.C.).

A

```
1       ATGGGCCCGGGGACCTCTCTTAGGCACCTGTTCTTGGTGCTGCAACTGGTGATGCTCCCG
1        M  G  P  G  T  S  L  R  H  L  F  L  V  L  Q  L  V  M  L  P

61      GCTGGCACTCAGGGAAAAGCAGTGGTGCTGGGTAAGGCAGGAGGCCAGGCAGAGCTGCCC
21       A  G  T  Q  G  K  A  V  V  L  G  K  A  G  G  Q  A  E  L  P

121     TGCCAGGCTTCCCAGAAGAAGAACATAGTCTTCAGCTGGAAAGATTCTTCCCAGTCCAAG
41       C  Q  A  S  Q  K  K  N  I  V  F  S  W  K  D  S  S  Q  S  K

181     ATTCTGGGGAGTCATAACTCCTTCTTGCACAAAGGTAACACTGAACTGAGCCGTCGAGTG
61       I  L  G  S  H  N  S  F  L  H  K  G  N  T  E  L  S  R  R  V

241     GAATCCAAAAGAAACCTGTGGGACCAAGGATCCTTTCCTCTGATCATCAAGAACCTCCAA
81       E  S  K  R  N  L  W  D  Q  G  S  F  P  L  I  I  K  N  L  Q

301     GTAACTGACTCGGGGACTTATACCTGTGAAGTGGATAGCAAGAAGCTCGAGGTGGAACTG
101      V  T  D  S  G  T  Y  T  C  E  V  D  S  K  K  L  E  V  E  L

361     AAGGTGTTCGGACTGACTGCCAGCTCGGACACCCGTCTACTGCTGGGACAGAGCCTGACC
121      K  V  F  G  L  T  A  S  S  D  T  R  L  L  L  G  Q  S  L  T

421     TTGACTTTGGAGAGTCCCTCTGGGAGCAACCCTTCTGTGCAGTGGAAGGGTCCAGGGAAT
141      L  T  L  E  S  P  S  G  S  N  P  S  V  Q  W  K  G  P  G  N

481     AATAGGAAGGAAGAACTCAAGAGCCTGTCACTGGCCCAGGTGGGGCTGCAGGACAGTGGT
161      N  R  K  E  E  L  K  S  L  S  L  A  Q  V  G  L  Q  D  S  G

541     ACCTGGACGTGCACTATCTCCCAGAGCCAGCAGACACTGGAGATCAAAATACCCATCGTG
181      T  W  T  C  T  I  S  Q  S  Q  Q  T  L  E  I  K  I  P  I  V

601     GTGCTGGCCTTCCAGAAGGCCCCCGAAACAGTCTATGTGAAGGAGGGGGAGCAGGCGGAG
201      V  L  A  F  Q  K  A  P  E  T  V  Y  V  K  E  G  E  Q  A  E

661     TTTTCCTTCCCACTCACCTTCGAGGATGAAAATCTGAGTGGGGAGCTGACTTGGCAGCAG
221      F  S  F  P  L  T  F  E  D  E  N  L  S  G  E  L  T  W  Q  Q

721     GCGAACAAGGATTCTTCCTCCCAATCCTGGGTCACCTTCACCCTGAGGAACAGAGAGGTG
241      A  N  K  D  S  S  S  Q  S  W  V  T  F  T  L  R  N  R  E  V

781     AAGGTGAACAAGACTCACAAGGACCTCAAGCTCCGTGTGGAGGAGAGGCTACCGCTGCGT
261      K  V  N  K  T  H  K  D  L  K  L  R  V  E  E  R  L  P  L  R

841     CTCACTCTGCTGCGGACCTTGCCTCAGTACGCGGGTTCTGGAACCCTGACCCTGGATCTC
281      L  T  L  L  R  T  L  P  Q  Y  A  G  S  G  T  L  T  L  D  L

901     TCCAAGGGGAAGCTGCATCAGAAAGTGAACCTCGTGGTGATGAGAGTGACTAAGTCCCCA
301      S  K  G  K  L  H  Q  K  V  N  L  V  V  M  R  V  T  K  S  P

961     AACAGTCTGACCTGTGAGGTGCTGGGGCCCAGCCCCCCAAGGCTGACCCTGAACTTGAAG
321      N  S  L  T  C  E  V  L  G  P  S  P  P  R  L  T  L  N  L  K

1021    CTGGGGAACCAGAGTATGAAGAGCTCAAATCAGCCAAAGTTGGTGACAGAGCCGGAACCC
341      L  G  N  Q  S  M  K  S  S  N  Q  P  K  L  V  T  E  P  E  P

1081    AAGGCTGGGATGTGGCAGTGTCTGCTGAGTGACCAGGGCAAAGTCCTGCTGGAATCCAAG
```

```
361     K A G M W Q C L L S D Q G K V L L E S K

1141    ATCGAGGTCTTGCCGTCAGAGTTCATCCAGGCCTGGCCGATGCTCCTGCCCATGGTGTTG
381     I E V L P S E F I Q A W P M L L P M V L

1201    GGGGGAATCGCAGGCCTAGCGCTTCTCACTGGCTCCTGCATCTTCTGTGTTAAATGCTGG
401     G G I A G L A L L T G S C I F C V K C W

1261    CACCGCAGGCGCCAGGCAGAACGGATGTCTCAAATCAAGAGGCTCCTCAGTGAGAAGAAG
421     H R R Q A E R M S Q I K R L L S E K K

1321    ACCTGCCAGTGCCCCCACCGTCTCCAGAAGACCCACAGTCTCACCTGA
441     T C Q C P H R L Q K T H S L T *
```

B

```
1       ATGTCGGCAGTGGTCCCAGGGGCCAGGAGGATGGAGCCAAGCTTGCTGATGTGGAGGTTC
1       M S A V V P G A R R M E P S L L M W R F

61      TTCGTATTCATCGTGGTACCTGGCTGCGTGACAGAGGCTTGTTATGATGACCCTCCGAGA
21      F V F I V V P G C V T E A C Y D D P P R

121     CTCAGAAACGCCATGTTCAAGGCCCTCAGGTACGAGGTGGGCACCATGATAAACTGCGAC
41      L R N A M F K A L R Y E V G T M I N C D

181     TGCAAGGCCGGCTTCCGCAGGGTGTCGGCCGTCATGCGCTGCGTGGGGGACTCCAGCCAC
61      C K A G F R R V S A V M R C V G D S S H

241     TCTGCCTGGAACAACAGATGCTTCTGCAACAGCACCTCCCCTGCTAAGAACCCAGTAAAA
81      S A W N N R C F C N S T S P A K N P V K

301     CCAGTTACTCCTGGATCCGAAGAACAGAGGGAGAGAAAACCCACAGATGCACAGAGCCAA
101     P V T P G S E E Q R E R K P T D A Q S Q

361     ACGCAGCCTCCGGAGCAAGCTGACCTTCCAGGTCACTGCGAGGAACCACCGCCATGGGAA
121     T Q P P E Q A D L P G H C E E P P P W E

421     CATGAACGTGAACCTTTGAAGAGAGTCTATCATTTCACGCTGGGGCAGACGGTTCACTAC
141     H E R E P L K R V Y H F T L G Q T V H Y

481     CAGTGTGCCCAGGGATTCAGGGCCCTACACACCGGTCCTGCTGAAAGCACCTGCACGATT
161     Q C A Q G F R A L H T G P A E S T C T I

541     ATCCACGGGGAGATGAGGTGGACCAGGCCCAGGCTCAAGTGCATAAGTGAAGGGGCGAAC
181     I H G E M R W T R P R L K C I S E G A N

601     AGTCAGGCTCCAGATGAAGCAGAGCCTCCGGAGAGCACGGAAGCTCCACCTGGGAGTGGA
201     S Q A P D E A E P P E S T E A P P G S G
```

661 ACTTTCTTAACAACCAGGACGGCAGGGACCACAGATTTCCAGAAGCCCACACGCGTGGTT
221 T F L T T R T A G T T D F Q K P T R V V

721 GCAACGCTGGATACGTTCATATTTACCACTGAGTACCAGATCGCAGTGGCCGGCTGCATC
241 A T L D T F I F T T E **Y Q I A V A G C I**

781 CTCCTGCTCTCCAGCGTCCTCCTCCTGAGCTGCCTCACGTGGCAGCGGAGATGGAAGAAG
261 **L L L S S V L L L S C L T W Q R R W K K**

841 AACAGAAGGACAATCTAG
281 **N R R T I ***

Supplemental figure S1.

Structural analysis of CD4 and CD25 genes in goats.

A, CD4 coding sequence region. B, CD25 coding sequence region. Signal peptide (red), extracellular region (black), transmembrane region (purple), intracellular region (green).

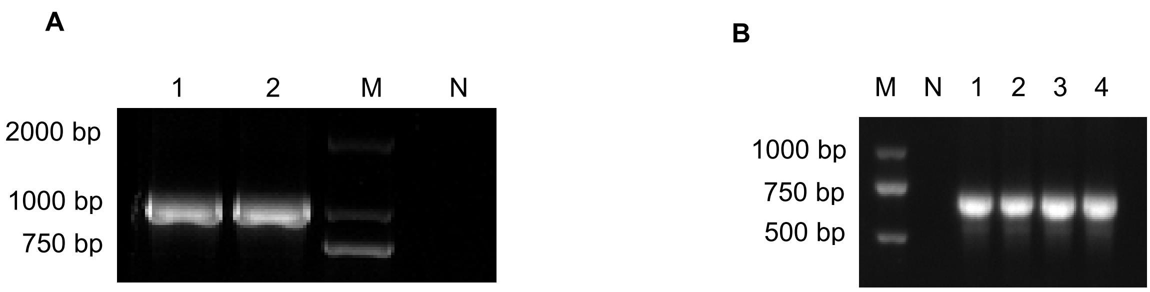

Supplemental figure S2.

Colony PCR analysis of the recombinant CD4 and CD25 plasmid.

A, Pcr results of recombinant plasmid CD4-PGX-6P-1. B, Pcr results of recombinant plasmid CD25-PET32a. M, DL2000 DNA marker. N, Negative controls. Lane 1-2, two different monoclonal colonies(A). Lane 1-4, four different monoclonal colonies(B).

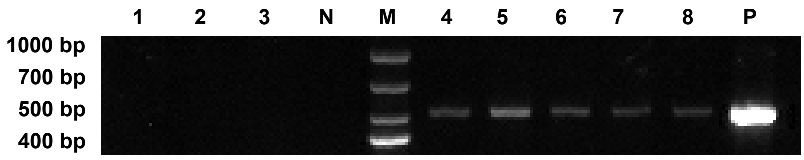

Supplemental figure S3.

Analyze B2L gene of Orf virus using samples of goat lip tissues.

M, DL2000 Maker; N, negative control; P, positive control; lane 1-3, healthy goat lip tissues; lane4-8, Orf virus-infected goat lip tissues.

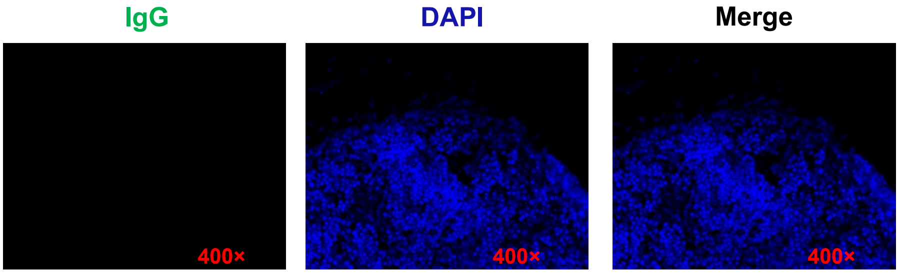

Supplemental figure S4.

Results of immunofluorescence homologous control antibody staining of goat thymus tissue. Homologous control antibodies do not stain.

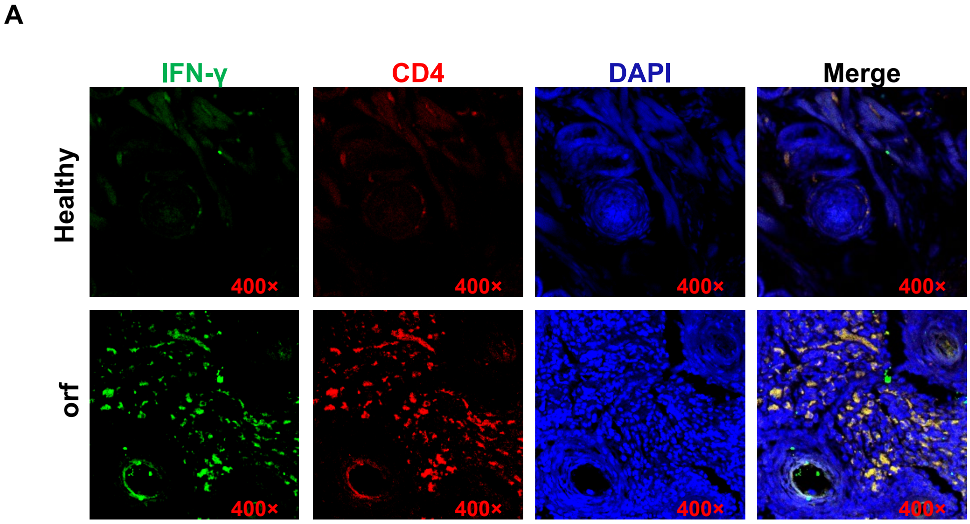

B

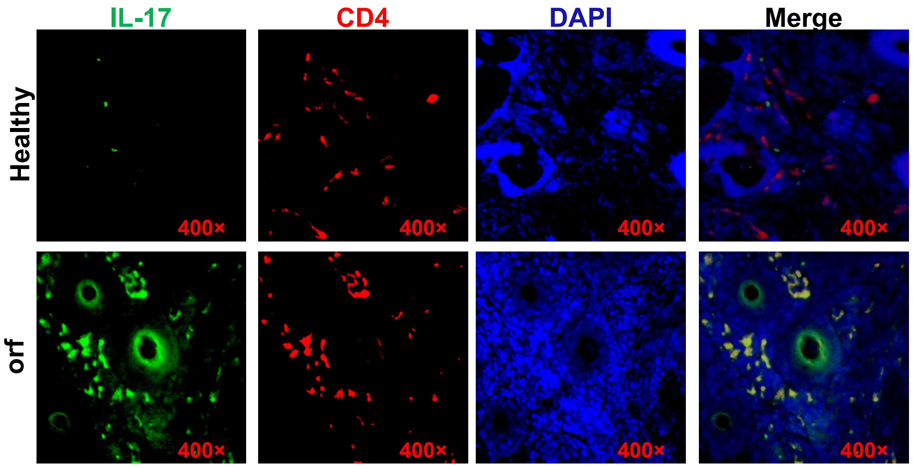

Supplemental figure S5.

Detection of Th1 and Th17 cells in goat lip tissue

A, Immunofluorescence staining of the lip tissues in healthy and Orf virus-infected goat using mAb D12 and IFN-γ mAb, Nuclear staining with DAPI. Th1 cells accumulated in the lip tissue of Orf virus-infected goat.

B, Immunofluorescence staining of the lip tissues in healthy and Orf virus-infected goat using mAb D12 and IL17 mAb, Nuclear staining with DAPI. Th17 cells accumulated in the lip tissue of Orf virus-infected goat.
